# Phenotype and function of peripheral blood γδ T cells in HIV infection with tuberculosis

**DOI:** 10.3389/fcimb.2022.1071880

**Published:** 2022-12-23

**Authors:** Shi Zou, Yanni Xiang, Wei Guo, Qi Zhu, Songjie Wu, Yuting Tan, Yajun Yan, Ling Shen, Yong Feng, Ke Liang

**Affiliations:** ^1^ Department of Infectious Diseases, Zhongnan Hospital of Wuhan University, Wuhan, China; ^2^ Wuhan Research Center for Infectious Diseases and Cancer, Chinese Academy of Medical Sciences, Wuhan, China; ^3^ Department of Intensive Care Medicine, Yichang Central People's Hospital, Yichang, China; ^4^ Department of Pathology, Zhongnan Hospital of Wuhan University, Wuhan, China; ^5^ Department of Pathology, School of Basic Medical Sciences, Wuhan University, Wuhan, China; ^6^ Wuhan Pulmonary Hospital, Wuhan Institute for Tuberculosis Control, Wuhan, China; ^7^ Department of Nosocomial Infection Management, Zhongnan Hospital of Wuhan University, Wuhan, China; ^8^ Department of Microbiology and Immunology, Center for Primate Biomedical Research, University of Illinois College of Medicine, Chicago, United States; ^9^ Department of Medical Microbiology, Wuhan University School of Basic Medical Sciences, Wuhan, China; ^10^ Hubei Engineering Center for Infectious Disease Prevention, Control and Treatment, Wuhan, China

**Keywords:** HIV, tuberculosis (TB), γδ T cells, TNF-α, IL-17A

## Abstract

**Background:**

Although γδ T cells play an essential role in immunity against *Human Immunodeficiency Virus* (HIV) or *Mycobacterium tuberculosis* (MTB), they are poorly described in HIV infection with tuberculosis (TB).

**Methods:**

The phenotypic and functional properties of peripheral blood γδ T cells in patients with HIV/TB co-infection were analyzed compared to healthy controls and patients with HIV mono-infection or TB by direct intracellular cytokine staining (ICS).

**Results:**

The percentage of Vδ_1_ subset in HIV/TB group was significantly higher than that in TB group, while the decreased frequency of the Vδ_2_ and Vγ_2_Vδ_2_ subsets were observed in HIV/TB group than in TB group. The percentage of CD4^+^CD8^-^ Vδ_2_ subset in HIV/TB group was markedly lower than in TB group. However, the percentage of CD4^+^CD8^+^ Vδ_2_ subset in HIV/TB group was markedly higher than HIV group or TB group. A lower percentage TNF-α and a higher percentage of IL-17A of Vδ_2_ subset were observed in HIV/TB group than that in HIV mono-infection. The percentage of perforin-producing Vδ_2_ subset was significantly lower in HIV/TB group than that in HIV group and TB group.

**Conclusions:**

Our data suggested that HIV/TB co-infection altered the balance of γδ T cell subsets. The influence of HIV/TB co-infection on the function of γδ T cells to produce cytokines was complicated, which will shed light on further investigations on the mechanisms of the immune response against HIV and/or MTB infection.

## Introduction

Tuberculosis (TB), caused by *Mycobacterium tuberculosis* (MTB) infection, is the most common opportunistic infection and the major cause of death among people living with HIV (PLWH) (UNAIDS, 2019). TB harms the immune response to HIV infection, accelerating the progression from HIV infection to AIDS. HIV is also recognized as a crucial risk factor for developing active TB. Therefore, understanding the immunological characteristics in the process of HIV/TB co-infection is crucial for disease control.

γδ T cells are non-conventional T cells that express a T cell receptor (TCR) consisting of a γ and a δ chain with diverse structural and functional heterogeneity. Although the proportion of γδ T cells in total peripheral T lymphocytes is only 1-5%, their rapid cytokine production following activation plays an essential role in patients with tumors, autoimmunity, and infectious diseases (Halary et al., 2005; Zhang et al., 2006; Wu et al., 2014). Depending on the difference in δ chain, γδ T cells can be defined into two major subsets, including Vδ_1_ and Vδ_2_. Vδ_1_ subset, rare in peripheral blood, mainly resides in the epithelium of mucosal tissue and participates in respiratory and intestinal mucosal immunity (Wu et al., 2014). Vδ_2_ subset is predominately distributed in peripheral blood and represents a significant portion of γδ T cells (accounting for 90%), and the Vγ_2_Vδ_2_ (also known as Vγ_9_Vδ_2_) subtype is the primary circulating Vδ_2_ subset (Wu et al., 2014).

Both Vδ_1_ and Vδ_2_ subsets are altered after HIV infection. Previous reports have described an increased level of Vδ_1_ subset and decreased level and function of Vδ_2_ subset in chronically HIV-infected patients (Li et al., 2008; Zheng et al., 2011). Furthermore, Vδ_2_ subset is associated with disease progression in HIV infection and can inhibit active viral replication (Poccia et al., 1999; Li et al., 2008). γδ T cells can also enhance human immunity against MTB by secreting cytokines and promoting anti-TB immune response caused by other immune cells such as macrophages (Kawakami et al., 2004; Zhang et al., 2006). Similar to HIV infection, the frequency and function of Vδ_2_ subset is also decreased in TB patients (Agrati et al., 2011; Negash et al., 2018). Few evidences suggested that HIV/TB co-infection had additive effects on peripheral Vδ_2_ subset depletion and dysfunction (Rojas et al., 2005; Shao et al., 2008).

The frequency and function of γδ T cells are still poorly understood, especially in HIV/TB co-infection. By directed intracellular cytokine stain (ICS) (Yao et al., 2014; Zou et al., 2022) and flow cytometry, this study was performed to evaluate the phenotypes and function of γδ T cells in peripheral blood of HIV/TB co-infection patients (uninfected controls, patients with HIV or TB infection as control group).

## Material and methods

### Study population

A cross-sectional study was performed involving subjects (≥ 18 years old) divided into four groups from May 2018 to March 2020. (1) Healthy control (HC) group: healthy donors with no history of chronic inflammatory diseases and infection signs in at least 2 weeks before peripheral blood collection were recruited from the physical examination center in Zhongnan Hospital of Wuhan University. They all received tests of HIV antibody screening, chest X-ray, and Interferon Gamma Release Assay (IGRA) to rule out HIV and MTB infection. (2) TB group: individuals recruited from Wuhan Pulmonary Hospital with a confirmed diagnosis of TB by etiological or histopathological methods (smear or culture positive, and/or MTB DNA test positive, and/or Xpert MTB/RIF test positive, and/or histopathological evidence supporting TB) and HIV antibody negative. They were recruited from Wuhan Pulmonary Hospital. All patients had been excluded HIV infection and hadn’t received anti-TB therapy prior to enrollment. (3) HIV group: individuals recruited with confirmed HIV infection by HIV antibody confirmatory testing but excluding MTB infection by chest X-ray and IGRA. They were recruited from AIDS Clinical Guidance and Training Center, Zhongnan Hospital of Wuhan University All patients hadn’t received antiretroviral therapy (ART) prior to enrollment. (4) HIV/TB group: individuals recruited with confirmed HIV infection and TB by the above diagnostic methods. All patients were recruited from department of infectious diseases, Zhongnan Hospital of Wuhan University and hadn’t received ART and anti-TB therapy prior to enrollment.

### Samples collection and isolation of PBMCs

For all groups, 10ml blood was drawn once with an ethylene diamine tetraacetic acid (EDTA) tube. Peripheral blood mononuclear cells (PBMCs) were isolated from freshly collected EDTA coagulated blood by Lymph prep (Axis-Shield, Norway) with density gradient centrifugation. Cell pellets were treated with 5 ml RBC lysis buffer (Sigma-Aldrich) for 10 min, followed by washing once with 5% FBS-PBS. PBMCs were then counted and cryopreserved until the following step experiments.

### Antibodies and reagents

The following antibodies (Abs) were used for short-term culture or surface marker and intracellular cytokine staining for flow cytometry (all Abs were from Biolegend): anti-CD3-PerCP-cy5.5 (Clone UCHT1), anti-CD8-APC-Cy7 (Clone SK1), anti-CD4-PE-Cy7 (Clone RPA-T4), anti-Vδ_1_-APC (Clone REA173), anti-Vδ_2_-PE (Clone B6), anti-Vγ_2_-FITC (Clone 7A5), anti-TNF-α-PE (Clone MAb11), anti-IFN-γ-PE-Cy7 (Clone 4S.B3), anti-interleukin-17A (IL-17A)-PE-Cy7 (Clone BL168), anti-Granzyme A-PE (Clone CB9), anti-Perforin-APC (Clone dG9), anti-granulocyte macrophage colony-stimulating factor (GM-CSF)-APC (Clone BVD2-21C11). The isotype control mAbs were purchased from the related company, respectively. The reagents listed below were all commercial products: brefeldin A (GolgiPlug, BD Biosciences), Cytofix/Cytoperm, and Perm buffer (BD Biosciences). The isotype control mAbs were purchased from the related company, respectively.

### ICS assay and flow cytometry analysis

This procedure was done exactly as we described (Yao et al., 2014; Zou et al., 2022). In brief, PBMCs were incubated for 1h with the medium in the presence of CD28 (1mg/ml) and CD49d (1 mg/ml) mAbs in a 200 ml final volume in round-bottom 96-well plates at 37°C, 5% CO2, followed by a 5-h incubation in the presence of brefeldin A (GolgiPlug; BD Biosciences). At the end of the incubation, cells were washed once with 2% FBS-PBS and stained at room temperature for at least 15-30 min with surface marker Abs (CD3, CD4, CD8, Vδ_1_, Vδ_2_ and Vγ_2_). After the next 45 min permeabilization (Cytofix/Cytoperm; BD Biosciences), another 45 min for intracellular cytokine staining (granzyme A, perforin, IL-17A, GM-CSF, IFN-γ and TNF-α) was performed. Finally, cells were re-suspended in 2% formaldehyde and subjected to flow cytometry analysis. To ensure the specific immune staining in direct or indirect ICS, matched isotype IgG served as negative controls.

### Statistical analysis

Flow cytometric data were analyzed with FlowJo version 7.6.1 for Windows. Statistical analysis of data was performed using GraphPad Prism version for Windows. Data are presented as a median and interquartile range. Analysis of non-parametric data used the Mann-Whitney U test for comparison of the median. Non-parametric rank sum test was used for comparison between groups. Multiple linear regression was employed to identify CD4 count associated with the γδ frequency and subsets. All reported P values were 2-tailed, and a P-value of 0.05 or less was considered significant.

## Results

### General information of subjects

During the study period, 121 individuals (25 in HC group, 40 in TB group, 29 in HIV group and 27 in HIV/TB group) were evaluated and designated in different groups according to the rules described in the methods. Characteristics of these individuals are summarized in **Table 1**. There was no statistical difference in age and sex composition among all groups. The median CD4^+^ T lymphocyte count (CD4 count) in HIV group was significantly lower than the HC and TB group, and the HIV/TB group had a lower CD4 count than HIV group (**Table 1**). All individuals were tested for phenotypes of γδ T cells in PBMC (**Table 1**), and 85 individuals were tested for function analysis (**Table 2**).

**Table 1:** General information of subjects in the study of phenotype analysis.

Characteristic	Patient group				
	HC (n =25)	TB (n =40)	HIV (n =29)	HIV/TB (n =27)	P-value
Age, median years (range)	33 (25, 30)	37 (28, 44)	37 (30, 49)	38 (29,51)	0.18
Male, no. (%)	13 (52)	22 (55)	17 (59)	17 (63)	0.09
CD4 count (cells/μL), median	862 (711, 929)	712 (620, 896)	402 (317, 588)	77 (39, 149)	0.00

Data are n (%) or median (IQR). Data are for participants with HIV and without HIV included in this analysis.

**Table 2:** General information of subjects in the study of function analysis for function analysis.

Characteristic	Patient group				
	HC (n =11)	TB (n =34)	HIV (n =18)	HIV/TB (n =22)	P-value
Age, median years (range)	31 (26, 38)	37 (27, 49)	35 (28, 47)	39 (33,51)	0.22
Male, no. (%)	7 (64)	19 (56)	11 (61)	15 (68)	0.12
CD4 count (cells/μL), median	861 (719, 982)	681 (579, 892)	371 (318, 569)	69 (40, 151)	0.00

Data are n (%) or median (IQR). Data are for participants with HIV and without HIV included in this analysis.

### The lower proportion of circulating Vδ_2_ subset in HIV/TB group

**Figure 1:**
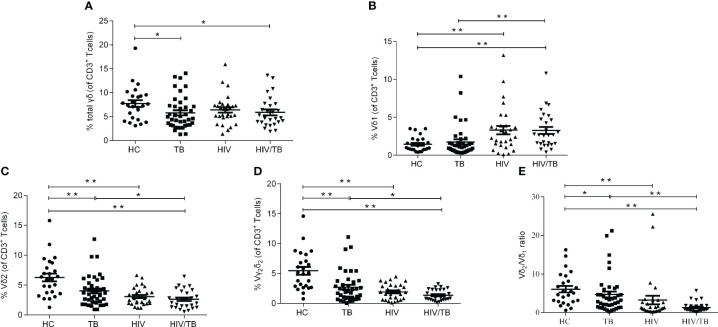
HIV or TB altered the balance of γδ T cell subsets respectively. **(A)** Percentages of γδ T cells in CD3^+^ T cells; **(B)** Percentages of Vδ_1_ subset in CD3^+^ T cells; **(C)** Percentages of Vδ_2_ subset in CD3^+^ T cells; **(D)** Percentages of Vγ_2_Vδ_2_ subset in CD3^+^ T cells; **(E)** Ratios of Vδ_2_/Vδ_1_. P-value calculated using the Mann–Whitney U test. Statistically significant differences between the groups were indicated as follows: *P<0.05 and **P<0.01. Those that were not statistically significant were not marked intra group or inter group.

To characterize the dynamics in γδ T cells during the course of HIV/TB co-infection, the percentage of γδ T cells (The sum of the percentage of Vδ_1_ subset and the percentage of Vδ_2_ subset), Vδ_1_ subset, Vδ_2_ subset and Vγ_2_Vδ_2_ subset in T lymphocytes were examined in HC, TB, HIV and HIV/TB groups. The flow cytometric gating strategy is illustrated in **Supplement Figure 1**. For the percentage of γδ T cells in T lymphocytes, except HC group, no significant difference was found between the HIV/TB and other groups (**Figure 1A**). The percentage of γδ T cells in TB was significantly higher than that in HC group (**Figure 1A**). As shown in **Figure 1B**, the percentage of Vδ_1_ subset between TB and HC group did not show statistical significance. In contrast, the percentage of Vδ_1_ subset in HIV and HIV/TB groups were significantly higher than that in HC and TB groups, respectively.

Vδ_2_ subset is the most critical functional subset of γδ T cells in peripheral blood. As shown in **Figures 1C**, compared with the HC group, the TB, HIV and HIV/TB groups all had significantly lower fractions of Vδ2 subset. Compared to TB group, the percentage of Vδ2 subset in HIV/TB group was significantly lower (**Figure 1C**). The results of the ratios of Vγ_2_Vδ_2_ subset and Vδ_2_/Vδ_1_ were similar to Vδ_2_ subset (**Figures 1D, E**). No significant difference of the percentage of Vδ2 subset or Vγ2Vδ2 subset was found between the HIV/TB and HIV groups. These results suggested that the damage of Vδ_2_ subset was prominent in HIV or MTB infection and HIV and MTB co-infection took it further.

### CD4^+^CD8^-^ Vδ_2_ subset survived from HIV infection but not HIV/TB co-infection

**Figure 2:**
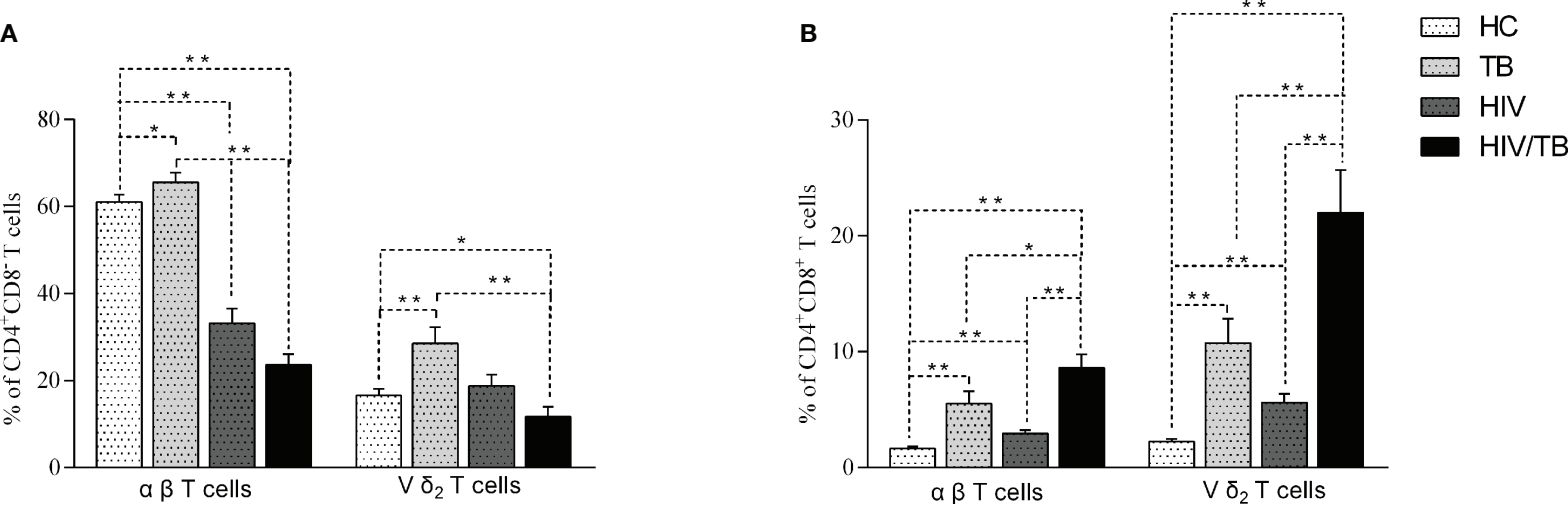
Comparison of the CD4^+^CD8^-^ **(A)** and CD4^+^CD8^+^ **(B)** Vδ_2_ subset and αβ T cells in each group. P-value calculated using the Mann–Whitney U test. Statistically significant differences between the groups were indicated as follows: *P<0.05 and **P<0.01. Those that were not statistically significant were not marked intra group or inter group.

The expression of CD4 or CD8 on αβ (CD3^+^ ${\text{Vδ}}_{1}^{-}{\text{Vδ}}_{2}^{-}$ T cells) and Vδ_2_ subset was analyzed in different groups. The percentage of CD4^+^CD8^-^ αβ and CD4^+^CD8^-^ Vδ2 T cells in TB group were significantly higher than in the HC group (**Figure 2A**). Compared with HC group, the percentage of CD4^+^CD8^-^ αβ T cells in HIV group was significantly decreased, while the percentage of CD4^+^CD8^-^ Vδ_2_ subset didn’t reduce in HIV group (**Figure 2A**). Furthermore, the percentage of CD4^+^CD8^-^ αβ and CD4^+^CD8^-^ Vδ_2_ T cells in HIV/TB group was markedly lower than that in TB group (**Figure 2A**).

We also analyzed the CD4^+^CD8^+^ αβ T cells and Vδ_2_ subset in the different groups. The results of CD4^+^CD8^+^ αβ T cells and Vδ_2_ subset were similar. Compared to TB group, the percentage of CD4^+^CD8^+^ αβ T cells and Vδ_2_ subset in HIV, TB and HIV/TB groups were significantly higher. Unexpectedly, the percentage of CD4^+^CD8^+^ αβ and Vδ_2_ subset in HIV/TB group were markedly higher than HIV and TB groups (**Figure 2B**). Our data suggested that HIV/TB co-infection resulted in a marked increase of CD4^+^CD8^+^ T cells.

### Percentage of cytokines producing Vδ_2_ subset in HIV/TB group

**Figure 3:**
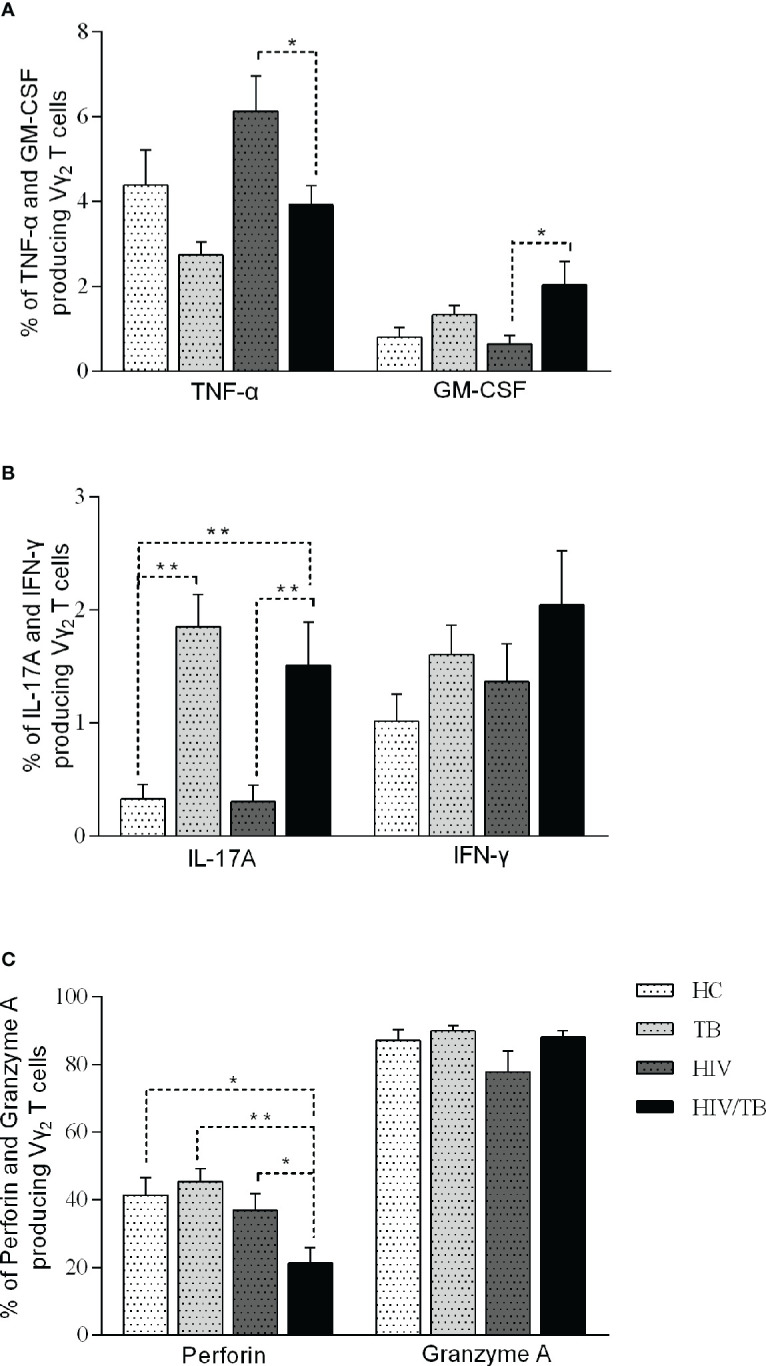
Function of Vδ_2_ subset among each group. Subjects that produce TNF-a and GM-CSF **(A)**, IL-17A and IFN-γ **(B)**, perforin and granzyme A **(C)** in each group are shown. P-value calculated using the Mann–Whitney U test. Statistically significant differences between the groups were indicated as follows: *P<0.05 and **P<0.01. Those that were not statistically significant were not marked intra group or inter group.

To further investigate the function of Vδ_2_ subset, we compared the cytokines production (TNF-α, GM-CSF, IL-17A, IFN-γ, Perforin and Granzyme A) in four groups (**Table 3**). All percentages represented the ratio of cytokine-secreting cells in Vδ_2_ subset. As shown in **Figure 3A**, there were no significant differences observed among the HC, TB, and HIV groups. In comparison with HIV/TB group, the percentage of TNF-α producing Vδ_2_ subset increased obviously in HIV group. On the contrary, the percentage of GM-CSF producing Vδ_2_ subset decreased obviously in HIV group compared to HIV/TB group.

**Table 3:** The frequency of γδ T cells produced different cytokines.

	HC group	TB group	HIV group	HIV/TB group
TNF-α	4.0 (2.2-5.4)	2.6 (1.3-3.8)	6.4 (3.3-8.8)**^b^**	3.9 (1.8-5.7)
IL-17A	0.1 (0.08-0.8)	1.3 (0.5-2.8)**^a^**	0.1 (0-0.3)**^b^**	0.8 (0-2.7)
IFN-γ	0.9 (0.4-1.5)	1.3 (0.5-2.0)	0.8 (0.3-2.3)	1.5 (0.2-3.4)
Granzyme A	86.0 (81.3-96.3)	93.8 (84.6-96.6)	86.5 (79.9-94.1)	91.4 (83.2-95.2)
Perforin	44.4 (30.5-53.6)	42.4 (26.6-64.1)**^b^**	35.5 (19.0-58.0)**^b^**	12.1 (2.1-42.8)
GM-CSF	0.7 (0.3-1.1)	1.1 (0.5-1.9)	0.4 (0.1-0.9)^b^	1.3 (0.4-2.1)

Data were shown as mean (interquartile range). **^a^** means the value of P is less than 0.05 as compared with healthy controls. **^b^** means the value of P is less than 0.05 as compared with HIV/TB group.

To IL-17, compared to HC group, the percentage of IL-17A producing Vδ_2_ subset in TB and HIV/TB groups were significantly higher. Similarly, the ratio of IL-17A producing Vδ_2_ subset in HIV/TB group was significantly higher than HIV group (**Figure 3B**). These data suggested that TB infection might disturb the production of IL-17A. However, the percentage of IFN-γ producing Vδ_2_ T cells did not alter among the four groups (**Figure 3B**).

The percentage of perforin produced in the Vδ2 subset was significantly lower in HIV/TB group than in the HC, HIV and TB groups (**Figure 3C**). However, there was no significant difference between HIV, TB and HC groups, respectively (**Figure 3C**). This indicated that HIV/TB co-infection suppressed perforin production in Vδ_2_ subset by certain mechanisms, but the mono-infection of these two pathogens did not show the depression of perforin in Vδ_2_ subset. However, the percentage of granzyme A producing Vδ_2_ T cells did not alter among the four groups (**Figure 3C**).

## Discussion

γδ T cells have been proven to have anti-viral capability by lysing HIV-infected cells *in vitro* (Gan and Malkovsky, 1996). They can also enhance human immunity against HIV or MTB by secreting cytokines with the stimulation of phosphoantigens (Poccia et al., 1999; Zhang et al., 2006). However, the phenotype and function of γδ T cells during HIV/TB co-infection were poorly understood. The aim of this study was to evaluate the roles of γδ T cells in the peripheral blood of patients with HIV/TB co-infection, thus offering new insights into the role played by these cells in immunity against MTB and HIV.

In agreement with previous reports that the destruction of γδ T cells was in accordance with HIV infection progression (Zheng et al., 2011; Li et al., 2015), the present study confirmed that HIV infection caused an increased percentage of Vδ_1_ subset and a decreased percentage of Vδ_2_ subset. Several studies proved that Vδ_2_ subset could inhibit and directly kill the intracellular MTB, thus preventing disease progression (Li et al., 1996; Dieli et al., 2001). In the present study, a lower proportion of Vδ_2_ subset was observed in TB group compared with HC group, which was consistent with other studies (Li et al., 1996; Carvalho et al., 2002). Whether the reduction of Vδ_2_ subset is a predisposing factor to the development of TB or is a consequence of MTB infection itself is still unknown. These results suggested HIV or TB infection could alter the balance of γδ T cells subsets (especially the decrease of Vδ_2_ subset), which may deteriorate the status of patients with HIV or MTB infection. Moreover, in HIV/TB co-infection patients, the proportion of Vδ_1_ and Vδ_2_ subset had a further increased and decrease, respectively. Vδ_1_ expansion may be linked to microbial translocation and may impact coinfection with several herpesviruses. Vδ_2_ subset plays an important role in controlling HIV replication and disease progression (Poccia et al., 1999; Li et al., 2008). The decrease of Vδ_2_ in the HIV/TB group may indicate a decrease in the body's ability to control HIV (Halary et al., 2005; Harris et al., 2010). The reduction in Vδ_2_ subset observed in our study may attribute to the presence of specific HIV and MTB ligands inducing a sustained activation of Vδ_2_ T cells, followed by a reduction in this cell subset by spontaneous and activation-induced apoptosis (Poccia et al., 1996; Li et al., 1998). Our results also indicated that HIV/TB co-infection might synergistically affect escaping the immune attack. In addition, some metabolites may influence the phenotype and function of γδ T cells. Various manipulations affecting isoprenoid metabolism can lead to the stimulation of Vγ_2_Vδ_2_ T cells (Wang et al., 2011). *In vitro*, the metabolic conversion of isopentenyl pyrophosphate (IPP) was blocked by zoledronate, resulting in the selective activation and expansion of Vγ_2_Vδ_2_ T cells (Roelofs et al., 2009; Wang et al., 2011). Moreover, mycobacterial metabolites, such as 3-Formyl-1-butyl-PP, were demonstrated to activate human γδ T cells (Belmant et al., 1999). How HIV/TB co-infection affects the metabolism is still unclear. Further studies on the function of Vδ_2_ subset need to explain why the HIV/TB co-infection progresses more quickly and is much more complicated to control than single pathogen infection from the immunological and metabolic aspect.

Most γδ T cells are CD4 and CD8 double negative, while only a tiny part express CD4 or CD8 molecules (Zheng et al., 2011). Our study revealed characteristics of CD4 or CD8 expression on γδ T cells in patients with HIV infection and TB. As is known, CD4^+^ T lymphocytes are attacked and destroyed by HIV. In our study, the proportion of CD4^+^CD8^-^ αβ T cells was significantly lower in HIV and HIV/TB group than that in HC and TB groups, respectively. However, the comparison between HIV and HC groups in our study revealed no significant difference in the percentage of CD4^+^CD8^-^ Vδ_2_ T cells. This result might raise doubt about that HIV possibly infecting γδ T cells by CD4 molecules and co-receptors (CXCR4 and CCR5) to cause the depletion of Vδ_2_ subset (Imlach et al., 2003; Spencer et al., 2013). This result illustrated that CD4^+^CD8^-^ Vδ_2_ T cells could survive during HIV infection and might be able to resist HIV infection but not HIV/TB co-infection. Recent studies suggested that double-positive cells (DP) T cells which accounted for only about 1%-2% of circulating humans are mature memory cells and may participate in the adaptive immune response against some infections (Overgaard et al., 2015; Zou et al., 2022). Our study described a significant increase in the percentage of DP T cells among Vδ_2_ subset and αβ T cells in the peripheral blood of HIV and TB group and incredibly high DP cells in HIV/TB co-infection group. These data showed that DP cells might play a certain role in the immune response of HIV or TB infection, particularly the co-infection of two pathogens.

Activated γδ T cells can secrete a variety of cytokines, including TNF-α, interleukin, IFN-γ, perforin, granzyme, and so on, which function as effector molecules in controlling infectious microorganisms (Li et al., 2008; Spencer et al., 2013). In this study, we investigated the function of γδ T cells in HIV and/or TB infection by analyzing cytokines produced by Vδ_2_ subset. TNF-α is the main component of the natural defense mechanism of the host. TNF-α can recruit monocytes, granulocytes and other innate immune cells to the location of infection and helps to form tuberculous granulomas in the infected tissues, which is beneficial to prevent the dissemination of TB bacteria in the body (Saunders et al., 2004). The levels of TNF-α also positively correlate with the progression of HIV infection (Benjamin et al., 2013; Vaidya et al., 2014), suggesting the critical role of TNF-α in HIV or/and TB. In the present study, we showed that the percentage of TNF-α producing γδ T cells was significantly lower in HIV/TB group compared to HIV group. However, there were no significant differences between the HC and TB group. Our data implied that HIV infection impaired the ability of γδ T cells to secrete TNF-α in active TB. Some studies also showed lower TNF production and cellular proliferation in MTB-specific peripheral T cells in HIV/MTB co-infection individuals than in individuals with HIV infection (Mendonca et al., 2007; Geldmacher et al., 2008), and HIV/MTB co-infected macrophages released fewer TNF-α than macrophages infected with only MTB (Kumawat et al., 2016). The reason might be that the expression of TNF-α in cells was down-regulated by HIV/MTB co-infection through some mechanism. Or the TNF-α-secreting cells may die through certain pathways in the case of HIV/MTB co-infection.

By inducing the expression of chemokines, recruiting monocytes and granulocytes and promoting the formation of granuloma, several studies demonstrated that IL-17 contributes to protective immunity in TB infection (Khader and Cooper, 2008; Torrado and Cooper, 2010). Nevertheless, some studies found that TB patients resistant to multiple drugs express high levels of IL-17 accompanied by severe tissue damage (Basile et al., 2011). Agree with another study (Peng et al., 2008), in the present study, we found that the percentage of IL-17A (referred to as IL-17) producing γδ T cells in TB patients was significantly higher than that in HC group, and there was a similar feature of change between HIV/TB and HIV patients. Our data not only further proved that γδ T cells might be involved in the immunity to MTB infection by secreting IL-17, but also suggests that HIV infection does not reduce the function of γδ T cells in producing IL-17. Considering the double-edged effect of IL-17, it is critical to elucidate further the mechanisms of IL-17 producing γδ T cells in the aspect of protective immunity and immunopathology during tuberculosis.

More studies show that effective cytotoxic T lymphocytes (CTL) responses require a coordinated expression of perforin and granzymes (Andersson et al., 1999; Chattopadhyay et al., 2009; Wu et al., 2014). A study describes that in TB, MTB-specific CD8^+^T cells express a low level of perforin but a relatively high level of granzyme A and B (Rozot et al., 2013). It is also reported that perforin^-^granzyme A^+^ CD8^+^ T cells had a weak capacity to kill target cells (Takata and Takiguchi, 2006). In our study, there was no significant difference in the percentage of γδ T cells secreting granzyme A among the four groups. However, the percentage of γδ T cells secreting perforin in HIV/TB group was significantly lower than that in HIV and TB group, and there was no significant difference between HIV and TB group and HC group. In addition, granzyme A secreted by Vδ_2_ subset has been found to induce TNF-α production in MTB-infected macrophages and inhibit the growth of intracellular MTB independently of perforin (Spencer et al., 2013). These results suggested that the anti-HIV and anti-TB effect of granzyme A and perforin secreted by Vδ_2_ subset was independent. Moreover, HIV/TB co-infection but not HIV or TB mono-infection could influence the ability of γδ T cells to secrete perforin.

This study has several limitations. First, the sample size was small. In addition, antigen-specific assays were not performed in our study. Although direct ICS represents the actual immune response in the human body, there is seldom such a high concentration of antigens in blood, and our results could not be compared with those of other studies using antigen-specific stimulation.

In conclusion, our results demonstrated a significant decrease in Vδ_2_ T cells in HIV/TB co-infection. Further studies on CD4^+^CD8^-^ Vδ_2_ T cells will be needed to clarify their ability to resist HIV infection. The influence of HIV and /or MTB on the function of γδ T cells to produce cytokines is very complicated, which will shed light on further investigations on the mechanisms of the immune response against HIV and/or MTB infection.

## Data availability statement

The original contributions presented in the study are included in the article/**Supplementary Material**. Further inquiries can be directed to the corresponding authors.

## Ethics statement

The studies involving human participants were reviewed and approved by Research and Ethics Committee of Zhongnan Hospital, Wuhan University, P. R. China (2016009-1K). The patients/participants provided their written informed consent to participate in this study.

## Author contributions

LS, YF, and KL conceived and designed this investigation. SZ, YX, and WG helped to design the scheme of the investigation and collected the original data. YT and QZ performed the experiments. YY and SW analyzed the data. SZ, YX, and KL contributed to the writing of the paper. All authors contributed to the article and approved the submitted version.
