# Supplementary figures and images for: Phenotype and function of peripheral blood γδ T cells in HIV infection with tuberculosis
Source: Front Cell Infect Microbiol. 2022 Dec 23;12:1071880. doi: 10.3389/fcimb.2022.1071880 (PMC9816428; doi:10.3389/fcimb.2022.1071880)

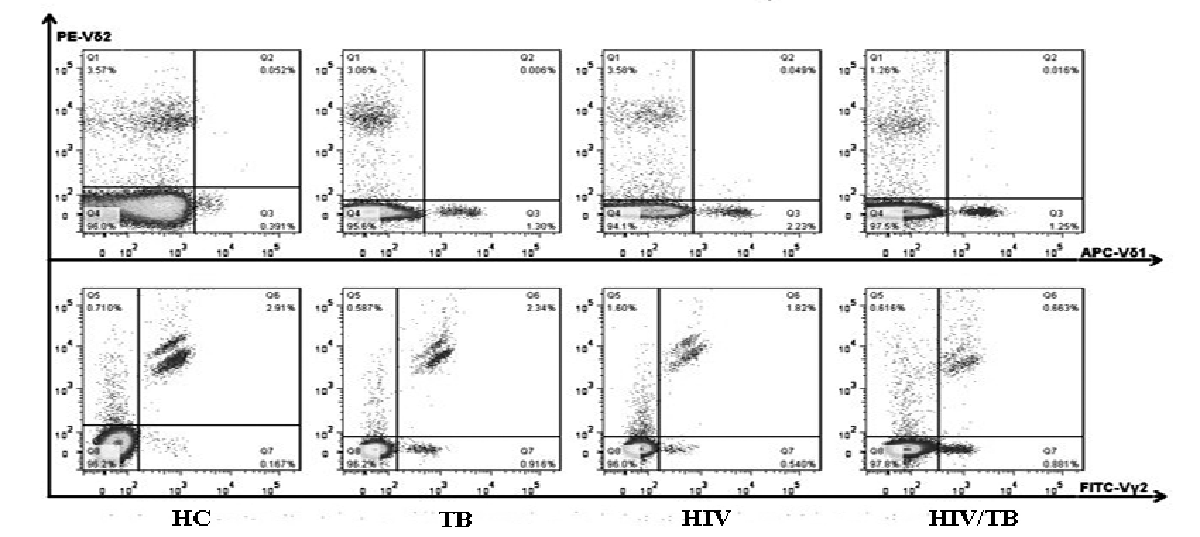

Supplement: Supplementary file 1 [file Image_1.tif]
